# An Overview of the H5N1 mRNA Vaccine Pipeline

**DOI:** 10.1111/irv.70113

**Published:** 2025-05-06

**Authors:** Daniele Focosi, Emanuele Nicastri, Fabrizio Maggi

**Affiliations:** ^1^ North‐Western Tuscany Blood Bank Pisa University Hospital Pisa Italy; ^2^ National Institute for Infectious Diseases “Lazzaro Spallanzani” Rome Italy

**Keywords:** H_5_N_1_, influenza virus, mRNA vaccines

Dear Editor,

An upcoming influenza virus A(H_5_N_1_) pandemic poses incredible challenges to the vaccine manufacturers community. At least 20 H_5_N_1_‐based vaccines have been already authorized for human use across the globe [1], but in animal models, they only provide partial immunity against the currently dominant 2.3.4.4b clade [2]. Of note, only Arepanrix, Aflunov, and Zoonotic Influenza Vaccine are updated on the currently circulating 2.3.4.4b clade, even considering that the two latter are based upon H_5_N_8_ and not H_5_N_1_ virus. In addition, 84% of the vaccine manufacturing capability relies over 11‐day‐old fertilized hen eggs (whose procurement is likely to suffer shortages during a bird flu pandemic), and only 16% relies over cell cultures (mostly MDCK cells). Further complicating scaling up, to spare antigen dose most vaccines require adjuvants (whose availability also represents a bottleneck), and it takes 4–6 months to have the first doses available since the WHO defines the candidate vaccine virus (CVV) [3]. There is then huge demand for alternative, faster vaccine manufacturing platforms.

In this regard, mRNA vaccines have built their success upon the COVID‐19 pandemic, when modified RNA (mod‐RNA) vaccines have been providing timely updates to the boosts, and many manufacturers are already testing in clinical trials mod‐RNA vaccines against different respiratory pathogens [4], including seasonal influenza [5]. A new generation of mRNA vaccines, named self‐amplifying mRNA (sa‐mRNA), has recently gained approval, with the promise of providing more doses, higher, and more durable immunogenicity [6]. In addition to the antigen cassette included in the mod‐RNA vaccines, sa‐mRNA constructs incorporate the replicase gene within the Alphavirus replicon [5], lowering the mRNA requirement per single vaccine dose from 30 μg down to 1 μg: A single manufacturing center can theoretically provide 8 billion doses of sa‐RNA vaccines [7]. The main hurdle for sa‐RNA vaccines is that uridine cannot be replaced by less immunogenic nucleosides, which rises the risk of both higher reactogenicity and immune responses against the replicase protein, which could impair the efficacy of boosts: Codon optimization is then required to minimize immunogenicity.

Table 1 summarizes the state of the art of H_5_N_1_ vaccine pipeline, which will likely translate into approvals in the upcoming months. It is clear that the majority of manufacturers is moving to mRNA platforms, and H_5_N_1_ sa‐mRNA vaccines are already in early clinical trials. Although the final CVV will likely be different from those currently tested in clinical trials, immunobridging will likely facilitate the authorization of updated vaccines. Moderna is also developing mRNA vaccines against pandemic candidates other than H_5_N_1_, such as H_10_N_8_ (VAL‐506440/mRNA‐1440) and H_7_N_9_ (VAL‐339851/mRNA‐1851).

**TABLE 1:** H_5_N_1_‐based vaccine pipeline.

Manufacturer	Country	Name	Vaccine type	Advancement
CSL	Australia	**CSL406** Influenza (H_5_N_1_) Vaccine	sa‐mRNA	Phase I (NCT06028347)
		**CSL400** (TIV) Trivalent influenza vaccine	sa‐mRNA	Phase I
Pfizer	USA	**pdmFlu**	mod‐RNA	Phase I (NCT06179446)
Moderna	USA	**mRNA‐1018** (against H_5_ and H_7_, and soon against 5 serotypes upon BARDA funding)	mod‐RNA	Phase I/II (NCT05972174)
CureVac/GSK	Germany	mRNA influenza A (H_5_N_1_) prepandemic vaccine candidate	(codon‐)optimized mRNA	Phase I/II (NCT06382311)
Sanofi	France	**SP0289**	mRNA	Phase I
Arcturus Therapeutics	USA	**ARCT‐2304** (aka LUNAR‐H_5_N_1_)	sa‐mRNA (STARR) into LUNAR LNP	Phase I (NCT06602531)

Cost per dose remains the main issue with mRNA vaccines, and again, sa‐RNA vaccines have the potential to lower this cost. H_5_N_1_mRNA vaccines for low‐and‐middle income countries have been sponsored by the WHO since July 2024 [8]. Outside pandemic settings, sa‐mRNA technology has the potential to translate the promise of universal influenza vaccines into reality.

## Author Contributions

**Daniele Focosi:** conceptualization, writing – original draft. **Emanuele Nicastri:** writing – review and editing. **Fabrizio Maggi:** writing – review and editing, conceptualization.

## Conflicts of Interest

The authors declare no conflicts of interest.

### Peer Review

The peer review history for this article is available at https://www.webofscience.com/api/gateway/wos/peer‐review/10.1111/irv.70113.

## References

[1] J. Taaffe , S. Zhong , S. Goldin , K. S. Rawlings , B. J. Cowling , and W. Zhang , “An Overview of Influenza H5 Vaccines,” Lancet Respiratory Medicine 3, no. 4 (2025): e20–e21. 10.1016/S2213-2600(25)00052-9 40090339

[2] D. W. Hawman , T. Tipih , E. Hodge , et al., “Clade 2.3.4.4b but Not Historical Clade 1 HA Replicating RNA Vaccine Protects Against Bovine H5N1 Challenge in Mice,” Nature Communications 16, no. 1 (2025): 655. 10.1038/s41467-024-55546-7 PMC11732985 39809744

[3] J. Taaffe , S. Goldin , P. Lambach , and E. Sparrow , “Global Production Capacity of Seasonal and Pandemic Influenza Vaccines in 2023,” Vaccine 51 (2025): 126839. 39970592 10.1016/j.vaccine.2025.126839 PMC11895838

[4] T. Troncoso‐Bravo , M. A. Ramírez , R. A. Loaiza , et al., “Advancement in the Development of mRNA‐Based Vaccines for Respiratory Viruses,” Immunology 173, no. 3 (2024): 481–496. 39161170 10.1111/imm.13844

[5] A. G. Lokras , T. R. Bobak , S. S. Baghel , F. Sebastiani , and C. Foged , “Advances in the Design and Delivery of RNA Vaccines for Infectious Diseases,” Advanced Drug Delivery Reviews 213 (2024): 115419. 39111358 10.1016/j.addr.2024.115419

[6] C. Chang , H. Patel , A. Ferrari , et al., “Sa‐mRNA Influenza Vaccine Raises a Higher and More Durable Immune Response Than mRNA Vaccine in Preclinical Models,” Vaccine 51 (2025): 126883. 39956088 10.1016/j.vaccine.2025.126883

[7] Z. Kis , C. Kontoravdi , R. Shattock , and N. Shah , “Resources, Production Scales and Time Required for Producing RNA Vaccines for the Global Pandemic Demand,” Vaccine 9, no. 1 (2021): 3. 10.3390/vaccines9010003 PMC7824664 33374802

[8] C. Terao , N. Bayoumi , C. A. McKenzie , et al., “Quantitative Variation in Plasma Angiotensin‐I Converting Enzyme Activity Shows Allelic Heterogeneity in the ABO Blood Group Locus,” Annals of Human Genetics 77, no. 6 (2013): 465–471. 23937567 10.1111/ahg.12034

