# Correction to “An Overview of the H5N1 mRNA Vaccine Pipeline”

**DOI:** 10.1111/irv.70256

**Published:** 2026-03-17

Focosi, D. , Nicastri, E. and Maggi, F. (2025), An Overview of the H5N1 mRNA Vaccine Pipeline. Influenza Other Respi Viruses, 19: e70113. 10.1111/irv.70113.PMC1205544940328677

The affiliation of Dr. Maggi F. and Dr. Nicastri E. was incorrect. This should have read: “National Institute for Infectious Diseases Lazzaro Spallanzani‐IRCCS, Rome, Italy”

We apologize for this error.

